# A rare case of prostato - symphyseal fistula after GreenLight photovaporization of the prostate

**DOI:** 10.1590/S1677-5538.IBJU.2018.0209

**Published:** 2019-04-01

**Authors:** Pablo Garrido-Abad, Manuel Ramírez-Sánchez, Luis García-Martín, Manuel Fernández-Arjona

**Affiliations:** 1 Department of Urology. Hospital Universitario del Henares, Coslada, Madrid, Spain

## DESCRIPTION OF CASE

**Figure 1:**
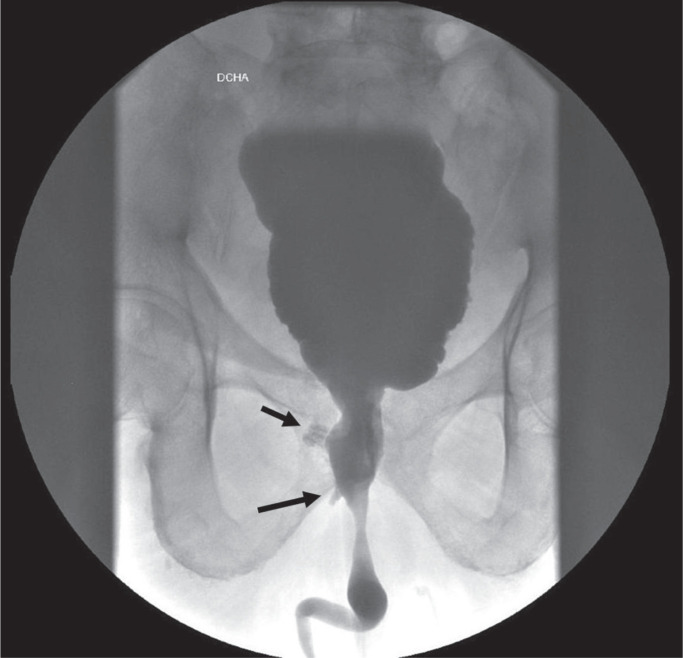
Voiding cystourethrography revealed small contained urethral leaks (black arrows).

**Figure 2:**
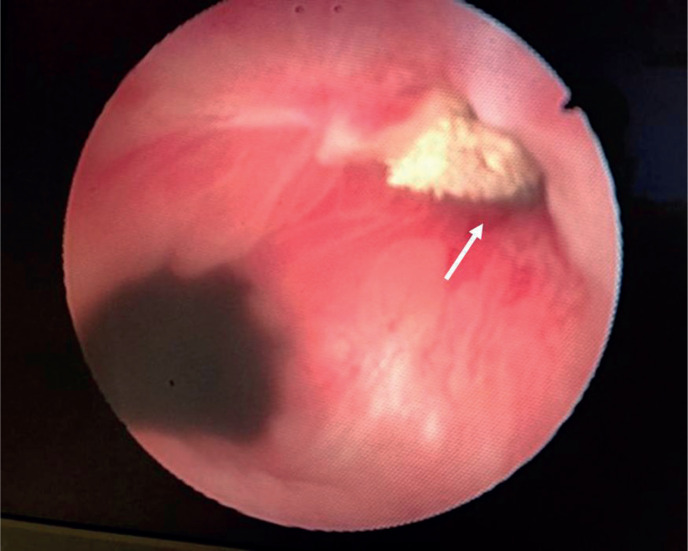
Endoscopic view showing an orifice of the fistula (white arrow) located in the anterolateral region of the prostatic fossa.

**Figure 3:**
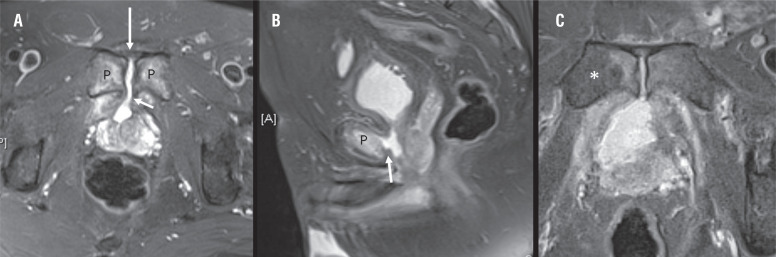
Pelvic MRI with fat suppression technique. (A) Axial T2 - weighted demonstrates the presence of urine (arrow) through the pubic symphysis (P). (B) Sagittal T2 - weighted shows anterior prostate - symphyseal fistula (arrow). (C) Axial T2 - weighted image displays edematous changes within both pubic bones, most markedly on right side (asterisk).

A 73 - year - old male patient was referred to us with worsened lower urinary tract symptoms and severe pubic pain. He had a history of benign prostate hyperplasia that was treated 2 years ago by GreenLight 180W XPS photovaporization of the prostate without intraoperative or immediate postoperative complications. At physical examination, selective pubic symphysis palpation was painful, exacerbated by ambulation. Voiding cystourethrography revealed a small contained urethral leak (Figure-1). Urethrocystoscopy showed an orifice with necrotic tissue at the 2 o'clock position of the prostatic fossa (Figure-2). The diagnosis of the anterior urinary fistula was confirmed by the presence of urine through the pubic symphysis (Figure-3A-B) in fat suppressed magnetic resonance imaging (MRI), with bone marrow edema within both pubic bones (Figure-3C). Conservative treatment was planned, including transurethral catheter placement for 3 months and 6 weeks of antibiotic therapy. However, due to worsening of the symptoms, a surgical repair was scheduled and is still pending.

Urosymphyseal fistulas occur when the integrity of the urethra is compromised, allowing urine leakage into the surrounding tissues, bacterial seeding of the pubic bone with the development of pubic osteomyelitis. Prostate - symphyseal fistula (PSF) is a rare complication of transurethral resection or photovaporization of the prostate. Fistulae may develop anteriorly (pubic symphysis) or posteriorly (rectum) and may originate from the bladder, prostate, or urethra. The exact incidence of anterior fistulae is unknown. A constellation of symptoms is associated with anterior urinary fistulae including osteitis pubis, osteomyelitis, recurrent urinary tract infections, pelvic pain, urine leakage, and / or sepsis (1). Hence, patient quality of life is severely impacted. Patients with small prostates (< 40 mL) may be at a higher risk for capsular perforation or thinning and subsequent development of osteitis pubis, osteomyelitis and PSF. Minimal use of high - power laser application in the anterior prostate tissue is recommended, particularly in small prostates (2). Computed tomography (CT) and MRI are the two imaging modalities of choice to establish a confirmed diagnosis. CT images at excretory phase enable the assessment of the presence of urine within the joint space in the case of PSF, whereas MRI is more sensitive to show inflammatory changes within the pubis or the adjacent soft tissues (3). Management of such complex patients requires a multidisciplinary approach. Patients who fail conservative management may need to undergo open repair of the fistulous tract, including omental, peritoneal or rectus abdominis flap interposition, and open radical retropubic prostatectomy.
